# Sphincter-Sparing Surgery in Patients with Low-Lying Rectal Cancer: Techniques, Oncologic Outcomes, and Functional Results

**DOI:** 10.1007/s11605-014-2528-y

**Published:** 2014-05-13

**Authors:** Liliana Bordeianou, Lillias Holmes Maguire, Karim Alavi, Ranjan Sudan, Paul E. Wise, Andreas M. Kaiser

**Affiliations:** 1 Department of Surgery, Massachusetts General Hospital, 15 Parkman Street, ACC 460, Boston, MA 02114 USA; 2 Department of Surgery, UMass Memorial Medical Center, Worcester, MA USA; 3 Department of Surgery, Duke University Medical Center, Durham, NC USA; 4 Department of Surgery, Washington University School of Medicine, St. Louis, MO USA; 5 Department of Colorectal Surgery, University of Southern California, Los Angeles, CA USA

**Keywords:** Rectal cancer, Sphincter preservation, Total mesorectal excision, Anterior resection, Intersphincteric resection, Local excision rectal cancer

## Abstract

**Background:**

Rectal cancer management has evolved into a complex multimodality approach with survival, local recurrence, and quality of life parameters being the relevant endpoints. Surgical treatment for low rectal cancer has changed dramatically over the past 100 years.

**Discussion:**

Abdominoperineal resection, once the standard of care for all rectal cancers, has become much less frequently utilized as surgeons devise and test new techniques for preserving the sphincters, maintaining continuity, and performing oncologically sound ultra-low anterior or local resections. Progress in rectal cancer surgery has been driven by improved understanding of the anatomy and pathophysiology of the disease, innovative surgical technique, improved technology, multimodality approaches, and increased appreciation of the patient’s quality of life. The patient with a low rectal cancer, once almost universally destined for impotence and a colostomy, now has the real potential for improved survival, avoidance of a permanent stoma, and preservation of the normal route of defecation.

## Historical Background

The modern era of rectal cancer surgery began with the description of the abdominoperineal resection (APR) by Miles in 1908. Prior perineal and sacral approaches (e.g., Kraske approach) to rectal cancer resection produced high rates of complications, cancer recurrence, poor quality of life (QOL), and poor overall survival. Miles’ operation reflected greater understanding of the natural history of rectal cancer that was based on postmortem examination of his patients after perineal resection. He observed cancerous implants in the pelvic peritoneum, mesorectum, and affected nodes of the left common iliac bifurcation. These findings led him to develop a “cylindrical concept” of the spread of rectal cancer to upward, downward, and lateral zones.^1^ The original operation included resection of the rectum, sigmoid, mesorectum, nodes of the iliac bifurcation, and a perineal component to include the anus and levator ani muscles. Postoperative mortality was high initially, but local recurrence decreased dramatically from 95 to 29 %.^2^ Subsequent improvements in antisepsis, anesthesia, and postoperative care improved survival significantly and made APR the gold standard operation for all rectal cancers. Over time, various modifications were made in patient positioning, perineal wound management, colostomy creation, use of drains, and adjuvant therapy, but the ultimate goal of the procedure, en bloc removal of the rectum with its lymphovascular supply, remained unchanged.

Surgical progress in the twentieth century limited the APR to cancers below the peritoneal reflection. Successful performance of anterior resection for cancers of the middle and upper rectum, as published by Claude Dixon in 1948^3^ led to the acceptance of this procedure and the creation of a “5 cm rule” from the dentate line—reserving APR for cancers below this level.

Improved understanding of cancer biology and surgical technology has led surgeons to accept ever smaller distal margins, which often translates into enhanced sphincter preservation rates. The oncologic and functional outcomes of preserving intestinal continuity, however, continue to be a matter of ongoing research and debate. Although an APR remains the appropriate approach for many low rectal tumors, the use of the procedure has steadily decreased over the last four decades, particularly in specialized centers.^4^ In his classic paper, Claude F. Dixon called the last 20 cm “the most controversial segment of the large intestine… It is for this region new procedures are constantly being advocated and interest in old ones is being rekindled”.^3^ Over 60 years later, his statement is still accurate, although perhaps most relevant now to the distal-most 5 cm of the rectum.

## Rationale for Sphincter-Sparing Techniques

Re-evaluation of the “5 cm rule” began in the 1980s and 1990s and the requisite length for distal resection margin (DRM) began to shrink. Paralleling the systematic introduction of radiation or chemoradiation for stage II and III cancers, the extent of the DRM was challenged by the recognition of the importance of circumferential resection margin (CRM), introduction of total mesorectal excision (TME) technique, and adoption of the circular stapler which facilitated low pelvic anastomoses. Williams et al. examined 50 potentially curative APR specimens and found 90 % had no or <1 cm distal intramural spread from the primary tumor.^5^ Furthermore, patients who did have intramural spread also had poorly differentiated primary tumors and ultimately succumbed to distant metastases. The authors concluded that rigid enforcement of the “5 cm rule” provided little oncologic benefit, especially in patients with adverse histologic features, while at the same time negatively impacted QOL metrics. Other studies confirmed that distal intramural spread of rectal cancer was uncommon and likely associated with high grade tumors and that survival was determined by metastatic disease rather than local recurrence.^6^ A retrospective review of 334 patients undergoing anterior resection with DRM <2, 2–5, or >5 cm found no difference in local recurrence (7.3, 6.2, or 7.8 %, respectively) or 5-year survival rates (69.1, 68.4, or 69.6 %).^7^ Similarly, Leo et al.^8^ found significant differences in survival and recurrence between patients with positive and negative DRMs, but no significant difference over 5 years between patients with negative DRMs <1 and >1 cm. Evidence in favor of smaller DRMs continued to accrue. A recent systematic review of 17 studies found no negative impact of DRM <1 cm or even <5 mm in terms of local recurrence or overall survival in patients with good risk tumors,^9^ supporting sphincter preservation even in very low tumors.

**Fig. 1:**
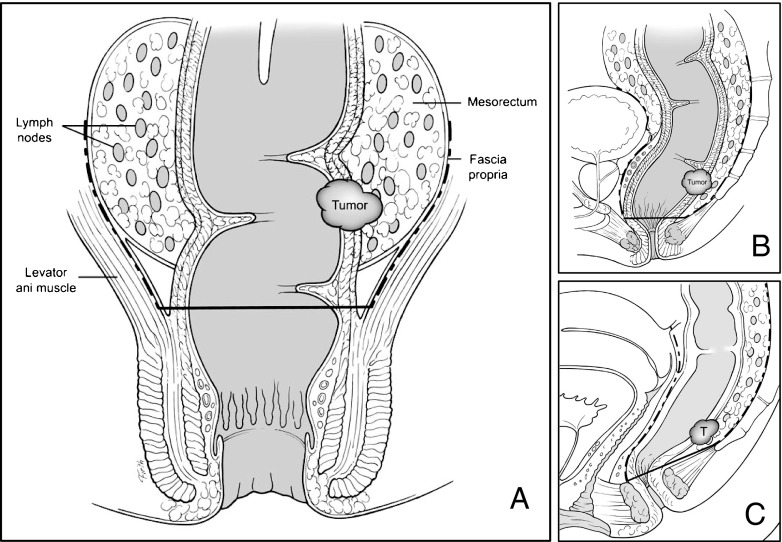
Appropriate planes for total mesorectal excision. **a** Anterior view demonstrating dissection plane between visceral mesorectal fascia and parietal fascia. **b** Lateral view of appropriate TME plane in the male. **c** Lateral view of TME dissection plane in the female

Reappraisal of the “5 cm rule” coincided with recognition of the importance of the CRM and the description of TME. Unacceptably high local recurrence rates after resection of rectal cancer led to investigations of the role of CRM and its correlation to local failure. Quirke and colleagues reviewed 52 rectal cancer specimens, finding lateral positive margins in one quarter and local recurrence in more than 80 % of those patients.^10^ Subsequent prospective studies confirmed the negative impact of positive CRM on recurrence and survival.^11^ Surgically, the CRM was addressed by TME. Championed by Heald, the concept of TME consisted of anatomically removing rectal cancers as a “tumor package” within the intact mesorectal compartment as defined by an embryonic plane between the parietal pelvic fascia and the visceral mesorectal fascia (Fig. 1).^12^ Adoption of TME resulted in decreased CRM positivity, decreased local recurrence, and increased survival for rectal cancer patients. The rise in TME rates for rectal cancers has coincided with a decrease in APR rates. Surgical dissection along the “holy plane” extends to the intersphincteric groove, allowing for dissection to the pelvic floor and creation of very low anastomoses.^13^ Adoption of TME in Sweden resulted in a fall in APR rates from 60 to 27 %, demonstrating the power of anatomical surgical technique in assuring an oncologically adequate dissection.^14^

Low anastomoses were facilitated not only by TME, but also by the introduction of the circular stapler. Initially developed in Russia, improvement and acceptance of the device allowed for low anastomoses to be performed more quickly and easily. In 1980, Heald reported a drop in the annual number of APRs performed from 27 to 4 in a single hospital following adoption of the circular stapler.^15^ Tolerance of smaller DRM, adoption of TME, and availability of circular stapling devices have dramatically impacted APR rates. These changes in approach to rectal cancer set the stage for new techniques for sphincter preservation in low rectal cancers including intersphincteric resection (ISR), perineal colostomy, transanal TME, and the Anterior and Perineal PlanE for ultra-low Anterior Resection of the Rectum (APPEAR) procedure (see below). Over the course of the twentieth century, the quality benchmark in rectal cancer surgery has progressed from the radical APR to the ability to preserve intestinal continuity and function while achieving excellent oncologic outcomes. APR can frequently be avoided if there is no evidence for direct or indirect invasion (e.g., fistula formation) of the pelvic floor and sphincter complex and in the hands of an experienced surgeon has a high probability of achieving a negative DRM and CRM.

## Techniques for Sphincter Preservation

### General Concepts

**Fig. 2:**
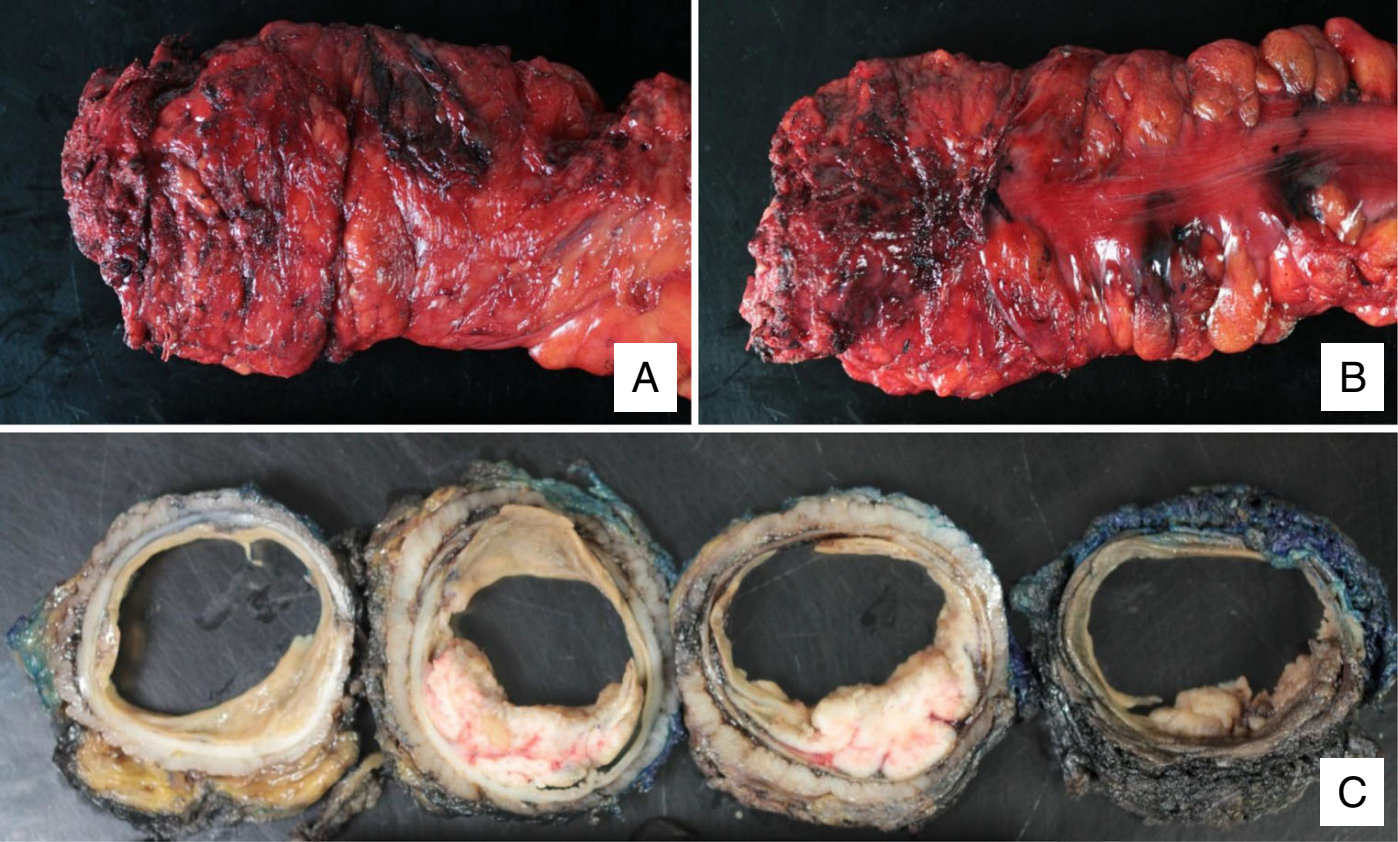
Total mesorectal excision specimen. **a**, **b** Gross view with intact mesorectum without “waisting” of the specimen. **c** Full thickness cross sections following fixation demonstrate a negative circumferential resection margin. Final pathology demonstrates a 1.5-cm T1 adenocarcinoma arising in a 5.5-cm tubulovillous adenoma

Successful resection of low rectal cancers is technically challenging. Important anatomic structures are seated deep within the pelvis and can be difficult to see and access. Patient characteristics, such as a narrow android pelvis or increased visceral fat, provide additional challenges. Regardless of the planned surgical approach, some assessments are broadly applicable. Determination of patient fitness for an operation and their preoperative continence may impact surgical options. Measurement of carcinoembryonic antigen (CEA) should be performed. Cross-sectional imaging of the chest, abdomen, and pelvis to assess for metastatic disease is standard, in addition to endoscopic clearance of the remainder of the colon. Review of the anatomy and structures involved by the tumor prior to the operation is essential. High quality rectal cancer-specific MRI scans for local tumor and nodal staging, in particular, can provide the surgeon with information on CRM and for planning neoadjuvant treatment and the resection. In experienced hands, endorectal ultrasound (EUS), as an alternative to MRI, can also provide local staging information, but is not as useful for assessing CRM. If a stoma is planned or considered, preoperative marking by the surgeon or enterostomal therapist should be performed for optimal placement. Use of infection-prevention measures and deep venous prophylaxis is standard for these major procedures. In most cases, the patient should be placed in stirrups intraoperatively to allow access to both the abdomen and perineum. Attention should be paid to positioning and padding the extremities in order to prevent compression injuries to the peroneal nerves and the brachial plexuses. Adequate lighting is crucial and head lamps for the surgeon and assistant can be very useful. Similarly, exposure is critical, and competent assistance, deep retractors such as the St. Mark’s, and long instruments are necessary. An extender for the electrocautery and an energy sealing device are useful for hemostasis deep in the pelvis. If a perineal portion is planned, synchronous abdominal and perineal dissection by two surgical teams allows manipulation of the tumor from above and below to achieve dissection in the correct plane. Alternatively, the perineal dissection can be completed in the prone position which may aid in exposure. The perineal dissection can be the most complex portion of the procedure, and transperineal or transrectal dissection (discussed below) may assist the surgeon in achieving adequate mesorectal resection for low tumors. Finally, the quality of the surgical resection is an important determinant of local recurrence and survival. Pathologic specimens should be evaluated and documented in a standardized fashion by both surgeon and pathologist, paying special attention to the intactness of the mesorectal envelope (Fig. 2).

### Total Mesorectal Excision

**Fig. 3:**
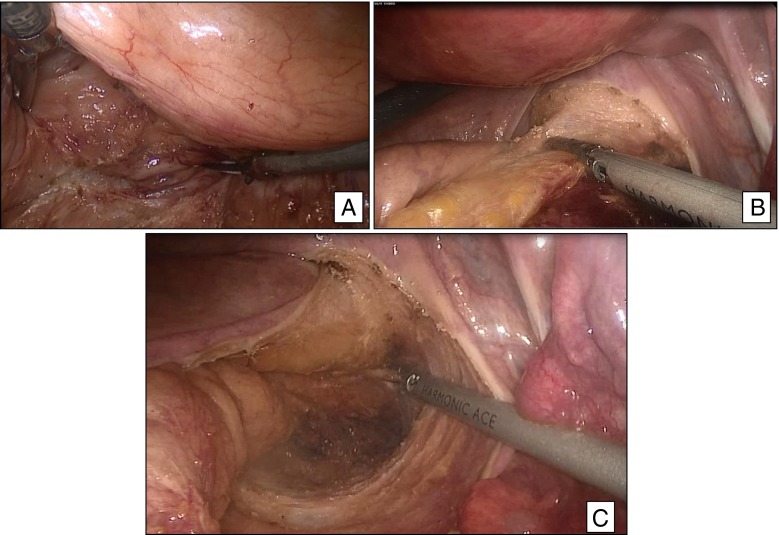
Total mesorectal excision. **a** Anterior dissection behind the seminal vesicles. **b** Anterior dissection in a female. **c** Dissection in the “holy plane” evidenced by an intact, shiny mesorectum

Total mesorectal excision exploits an embryologic avascular perimesorectal plane to extract a cylindrical specimen of rectum and mesorectum. Anatomical dissection under direct vision is carried out in this areolar plane, maintained and revealed by three-dimensional tension applied by the assistant. Preservation of nerves critical for normal sexual and bladder function is a hallmark of the technique. The technique of TME has been described in detail^16^ and is summarized briefly here. Following entry and exploration of the abdomen, mobilization of the splenic flexure, proximal ligation of the colon, and high ligation of the inferior mesenteric vessels, pelvic dissection commences and proceeds circumferentially. Posteriorly, dissection is carried past the tip of the coccyx, completed by sharp division of the rectosacral ligament. Lateral extension carries the plane between the superior hypogastric plexus and the mesorectum. As lateral dissection proceeds inferiorly, meticulous maintenance of a plane between the increasingly dense and adherent plexus and the mesorectum is required. Straying medially from the plane in this region may compromise the circumferential margin and produce bleeding from mesorectal vessels; straying laterally may injure the nerves of the hypogastric plexus and/or cause bleeding from the pelvic sidewall. Following the plane anteriorly, dissection proceeds through Denonvilliers’ fascia or the rectogenital septum between the mesorectum and prostate and seminal vesicles or posterior wall of the vagina (Figs. 1b, c and 3a, b). Again, care is required to avoid straying posteriorly compromising the resection margin or anteriorly to avoid injury to the inferior hypogastric plexus. Continued dissection circumferentially in this plane (Fig. 3c) leads to the intersphincteric plane and a clean muscle tube for distal transection.

Although never compared to traditional surgical approaches in a prospective, randomized fashion, TME demonstrates clear superiority in terms of local recurrence and survival as compared to historical controls. In 1998, Heald and colleagues reported their experience with 519 patients, few receiving adjuvant therapy. Their local recurrence rate was 8 % and cancer-specific survival was 66 % at 10 years.^17^ These results represented a substantial improvement over local recurrence rates of 30 % or higher in the literature at the time. Reproduction of similar results in other centers^18^ led to the acceptance of TME as the standard of care.

### Function After Low Anterior Resection

While TME (commonly in conjunction with chemoradiation) has had a significant impact on local recurrence rates of rectal cancer, patients frequently experience varying degrees of altered bowel function. Low anterior resection syndrome (LARS) includes multiple bowel symptoms, may be associated with urinary or sexual dysfunction, varies in severity, and may impact QOL. Patients report symptoms of urgency, incontinence, and difficult evacuation at rates of 12–45, 10–71, and 16–74 %, respectively.^19^ Some degree of improvement with time is the norm, but symptoms can persist as late as 15 years postoperatively.^20^ Postoperative factors contributing to the development of LARS include shortened intestinal length and a diminished rectal reservoir as higher volumes of more liquid stool are delivered to a smaller neorectum. Anorectal manometry reveals reductions in urgent volume, maximal tolerable volume, and rectal compliance.^21^ Other factors contributing to the development of LARS include damage to the sphincter complex or its innervation. Sympathetic nerves are at risk during high ligation of the inferior mesenteric artery (IMA), and parasympathetic nerves may be injured when the surgeon attempts to obtain wide negative CRMs. The levator ani nerve, arising from S3 and S4, runs on the superior surface of the pelvic floor, making it vulnerable to injury during dissection and potentially causing a dysfunctional pelvic floor postoperatively.^22^

Despite the increased focus on visualization and preservation of the pelvic autonomic nerve structures, symptoms consistent with pelvic floor dysfunction are common following TME. Of 178 participating patients with normal continence in the Dutch TME trial, 69 (39 %) had new fecal incontinence after rectal cancer treatment and 14 % had new onset combined fecal and urinary incontinence.^22^ These troubling results warrant further investigation. Although the percentage of patients with postoperative functional problems remains substantial, it is significantly reduced relative to functional outcomes reported in the past. Today, functional outcome is influenced through multiple factors, including the patient, (neo) adjuvant therapy (radiation and chemotherapy toxicity), surgical technique, and the method and level of anastomosis.

## Intersphincteric Resection

**Fig. 4:**
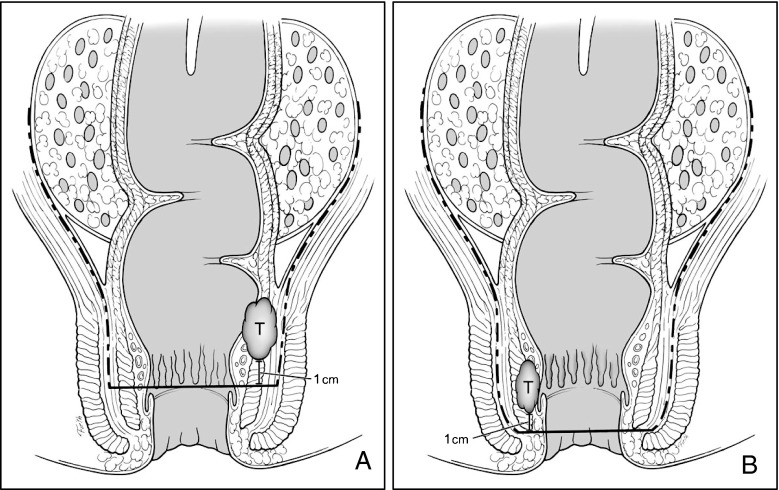
Appropriate planes for intersphincteric resection—TME plane created from above intersects intersphincteric plane. **a** Partial ISR; **b** complete ISR

**Fig. 5:**
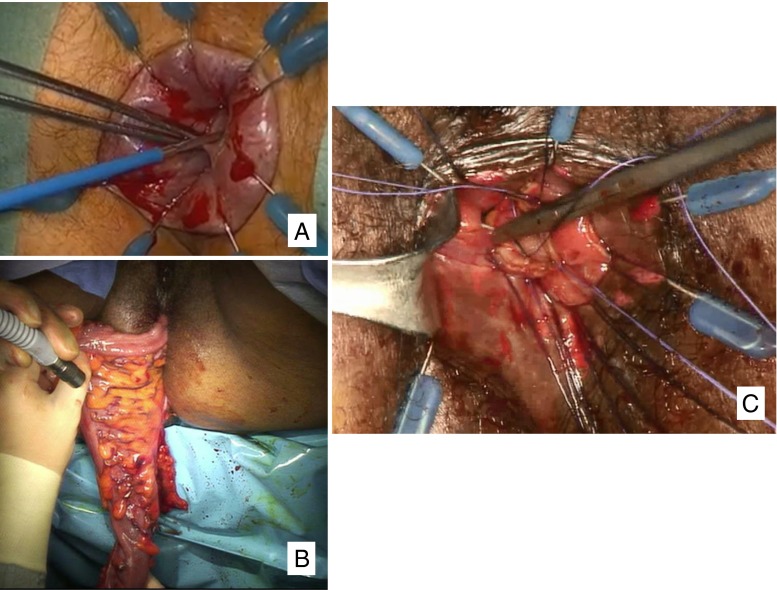
Complete intersphincteric resection. **a** Dissection begins at the dentate line. **b** The rectum is eviscerated through the anus after joining of the abdominal and perineal dissection planes. **c** Handsewn coloanal anastomosis

Schiessel and colleagues described intersphincteric resection (ISR) for rectal cancer in 1994.^23^ ISR exploits the plane between smooth and striated sphincters to achieve a balance between adequate oncologic resection and continence preservation (Fig. 4). Proctectomy and TME are combined with resection of all or part of the internal anal sphincter and creation of a handsewn transanal anastomosis (Fig. 5).

The operation is carried out by abdominal and perineal approaches. The abdominal approach consists of a proper TME as described previously. The perineal approach then allows for identification of the mass. Under direct vision, the surgeon transects the internal sphincter 1 cm below the tumor. The dissection is then carried cephalad to connect with the TME plane developed transabdominally. As with all TME procedures, a protective loop ileostomy is routinely constructed. Laparoscopic^24^ and robotic^25^ approaches have been described.

Intersphincteric resection is technically feasible because the internal sphincter is a continuation of the rectal wall (muscularis propria), which does not have mesorectum distal to the levator ani muscles. Oncological outcomes, as with all procedures, are dependent on proper patient selection. Oncological concern related to the ISR stems from the possibility that tumor extends beyond the intersphincteric plane into the external sphincter, thus leading to a positive radial excision margin—a circumstance that would likely require APR for cure. Large series of patients undergoing ISR have been published by American, European, and Asian groups.^26^ Recently, a review of 14 high quality reports including over 1,200 patients was published.^27^ An R_0_ resection was achieved in 97 % of patients with an average DRM of 1.7 cm and a negative CRM in 96 %. Mean oncologic outcomes were similar to initial reports but ranges varied widely. The overall rate of local recurrence was 6.7 %, but ranged from 0 to 23 %. Overall survival was 86.3 % (62–97 %) and disease-free survival was 78.6 % (69–87 %).

Given that ISR removes all or part of the internal anal sphincter, patients should be carefully informed in regard to morbidity and functional outcomes following this procedure. ISR should be reserved for young patients with strong preoperative sphincter pressures and long anal sphincters. An early study by Shissel et al. reported satisfactory continence but slightly higher recurrence rates.^23^ A follow-up study demonstrated a 30-day morbidity of 7.7 % with 9.4 % developing a late coloanal stricture. Local recurrence rates were low at 5.3 %, and mean overall survival was 126 months. Continence for flatus, liquid, and solid stool was maintained in 86 % of patients at long-term follow-up. Frequency of defecation was initially high, but dropped to slightly greater than 2/24 h after 6–12 months. Although continence results were acceptable, symptom-specific QOL indices were significantly lower in patients after ISR or coloanal anastomosis as compared to patients after anterior resection.^28^

Lack of a standardized assessment instrument and inconsistent reporting of functional outcomes in recent, larger series make consensus on functional outcome after ISR difficult. In a review of eight studies reporting outcomes, 11–63 % of patients reported fecal soiling and 30–86 % reported perfect continence^27^ with statistical assessment of the data showing wide confidence intervals. The most likely interpretation of the results is that functional outcome after ISR is highly variable. Suggested modifications to the procedure to improve continence include a coloplasty^29^ or creation of a colonic pouch. However, one study found the creation of a pouch to be less critical for postoperative continence than tumor level and height of the anastomosis.^30^ ISR, of necessity, interferes with the mechanisms of continence and does not generate perfect functional results. Difficulties include frequent defecation, stool fragmentation, and incontinence. In considering ISR, the surgeon and patient must consider the burden of these symptoms versus the alternative, a permanent ostomy following APR.

## APPEAR

**Fig. 6:**
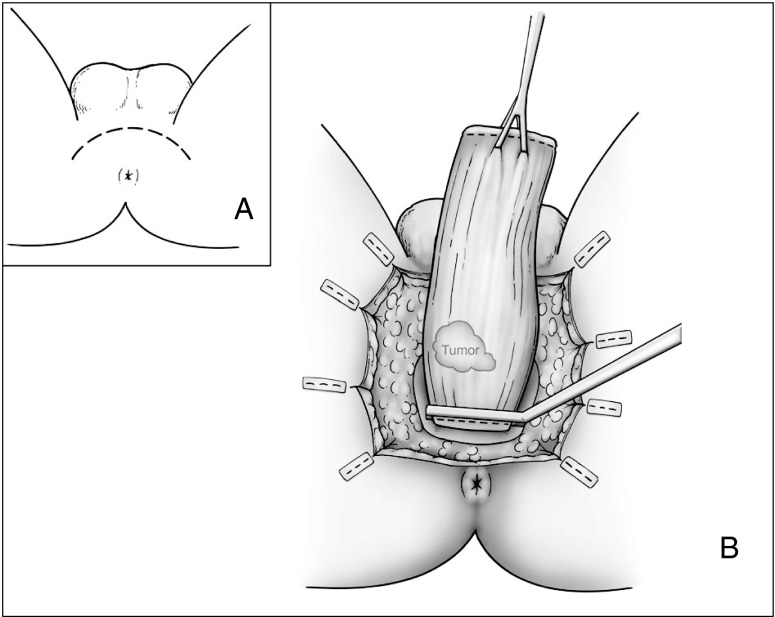
Perineal dissection in the APPEAR technique. **a** Perineal incision. **b** Evisceration of the specimen through the perineal wound after connection of abdominal and perineal dissection planes

The Anterior Perineal PlanE for Ultra-low Anterior Resection of the Rectum (APPEAR) technique is another recently described method allowing very low rectal resection of rectal cancer.^31^ A combined abdominal and perineal approach allows access to low, difficult to access rectum between the levator ani and the superior margin of the external anal sphincter. Following transabdominal mobilization of the rectum and transection of the rectosigmoid, a perineal wound is created and a rectovaginal/rectrourethral plane developed and carried upward to that created by the abdominal surgeon. This approach spares the anal sphincter entirely yet it allows the surgeon to dissect at least 2–3 cm lower than one could from above. The rectum is freed laterally and posteriorly from the perineal aspect and the specimen delivered through the perineum (Fig. 6). A straight coloanal or a pouch anastomosis and a protecting ostomy are created. An initial report of 14 patients (seven with rectal neoplasia) described no mortality but significant morbidity, with seven patients (50 %) developing perineal infection and fistulae.^31^ No local recurrences were appreciated, although one patient developed systemic disease. The authors found a median Wexner continence score of 6 (range 0–8) after ileostomy closure and good QOL measures.

Compared to ultra-low anterior resection, the APPEAR technique has the advantage of providing greater distal access to the rectum for mobilization and, compared to ISR, has the advantage of not disrupting the sphincters. However, a troublesome perineal wound is created, and thus far, data on oncologic outcomes are limited. Further time and study are required to evaluate the promise of this technique.

## Transanal TME

**Fig. 7:**
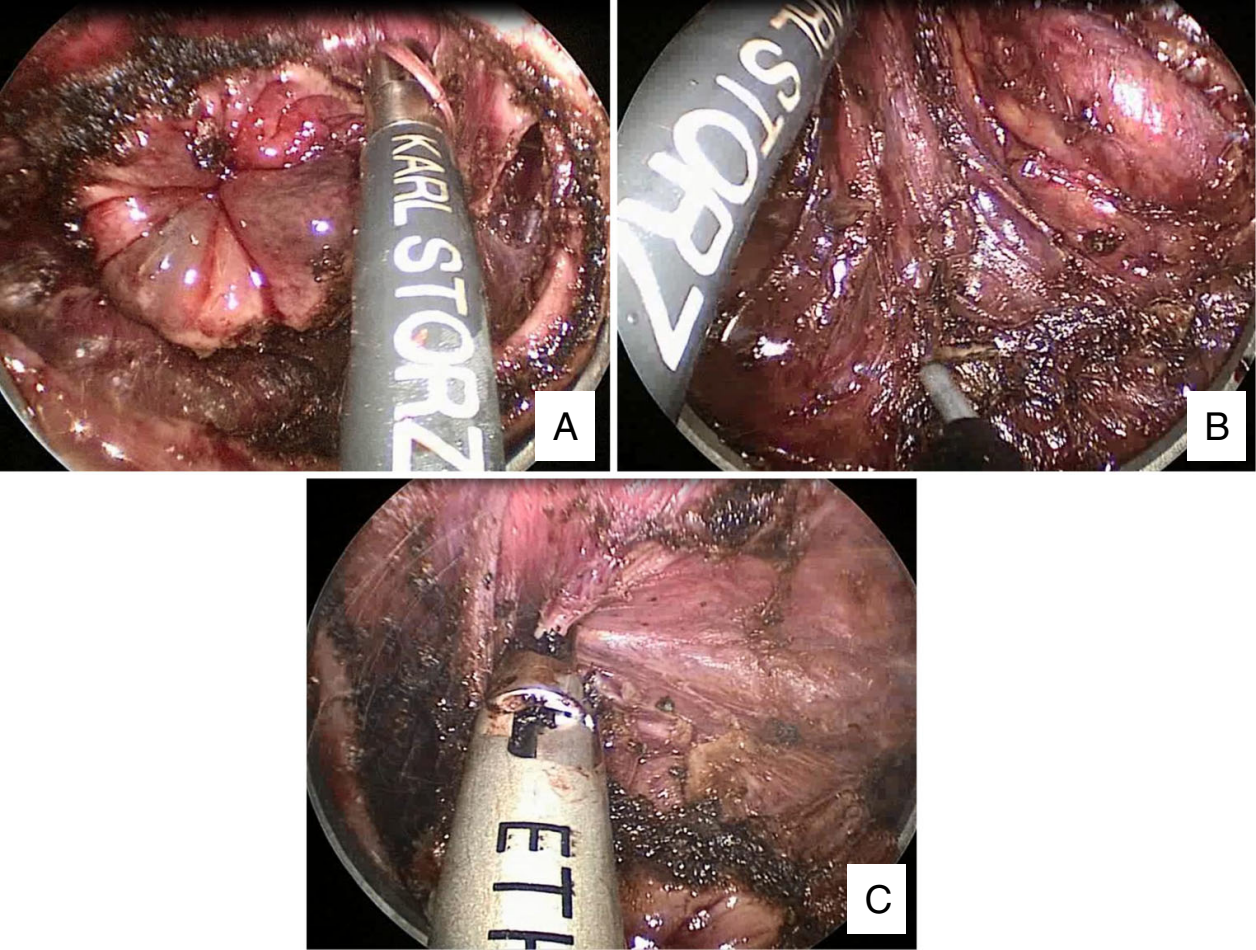
Transanal TME. **a** Dissection begins following the placement of a purse string distal to the tumor. **b** The posterior mesorectum. **c** Dissection posterior to the vagina

A second technique to improve perineal dissection and facilitate sphincter-sparing resection of ultra-low rectal tumors is transanal TME, a natural orifice transluminal endoscopic surgery (NOTES) approach to rectal cancer. International and US^32^ case series have been reported. Transabdominal laparoscopic dissection is carried out in standard fashion following TME principles. After placement of a purse-string suture in the rectum >1 cm distal to the tumor, perineal dissection is carried out transrectally via a multi-instrument port inserted in the anus (Fig. 7). The specimen is extracted and anastomosis created transanally. This approach also facilitates access to rectal “no man’s land,” but compared to the APPEAR technique, has the advantage of not creating a separate perineal wound. Report of 20 patients undergoing the procedure described an average DRM of 2.6 cm and CRM of 1.8 cm.^33^ In this series, 20 % of patients had complications: two with urinary retention, one with ileus, and one with dehydration. In total, 72 patients have been reported in the literature (reviewed in^34^), but no long-term oncologic outcomes are available. As with APPEAR, transanal TME has the potential to improve the perineal aspect of the dissection and achieve a sphincter-sparing R_0_ resection in more patients with low rectal cancer, but further study is necessary to evaluate long-term oncologic outcomes.

## Local Excision

### Selected Early Rectal Cancers May Be Managed with Local Excision

Local excision of rectal cancer may be performed as definitive therapy for selected early rectal cancers, in combination with chemoradiotherapy for more advanced tumors, or as a palliative procedure for patients unable to undergo transabdominal operation. Development of novel techniques such as transanal endoscopic microsurgery (TEM) and transanal minimally invasive surgery (TAMIS) underscores the enthusiasm for complete avoidance of transabdominal surgery for rectal cancer. However, the oncologic adequacy of these operations remains a matter of study and debate. There is a lack of prospective, randomized trials and published series vary in terms of patient selection, adjuvant therapy, surgical technique, and length of follow-up. Nonetheless, rates of local excision are increasing^35^ and reflect the interest of patients and surgeons in avoiding major, morbid operations for early stage disease.

### Patient Selection

Regardless of surgical technique, appropriate patient selection is the most critical and challenging element in achieving adequate oncologic outcomes with local excision. Ideally, tumors removed by local excision are node-negative, pT1 tumors with favorable histology, but none of those factors may be determined with certainty preoperatively. Patients selected for local excision should have small, mobile tumors and EUS or MRI results suggesting the tumor is node negative and confined to the bowel wall. Clinical exam and radiologic studies provide important but imperfect data. Meta-analysis of endorectal ultrasound found a sensitivity and specificity of 73.2 and 75.8 % for lymph node involvement^36^ and 87.8 and 97.8 %, respectively, for staging T1 tumors.^37^ MRI specificity was 75 % for tumor stage and 71 % for lymph node involvement.^38^ A thorough workup cannot completely confirm that a tumor will be definitively treated with local excision.

Postoperatively, pathological features determine whether or not the patient is likely to have had adequate surgical therapy or requires TME. Tumor characteristics including size, depth of invasion, and lymphovascular invasion are important determinants of local recurrence. Bach et al.^39^ analyzed 424 patients after TEM for rectal cancer and found that 93 % of patients with well to moderately differentiated pT1 tumors, <3 cm in diameter, without lymphovascular invasion, with an R_0_ resection were free of recurrence at 36 months; however, any violation of these qualities led to significantly increased rates of recurrence. The authors concluded that local excision ought to be considered compromise therapy or an excisional biopsy for patients not meeting these characteristics. Similarly, Borschitz et al. found that patients with high risk T1 tumors, <1 mm margins, tumor fragmentation, or R1 resection had 49 % 10-year survival as compared to 89 % among patients with low-risk tumors and R_0_ resection.^40^ The discovery of adverse pathologic characteristics after local excision should be followed by immediate radical resection, which may be performed without adverse oncologic outcome compared to primary radical resection.^41^ However, even with intensive surveillance, delaying TME until the clinical appearance of recurrence has been shown to lead to significantly worse rates of survival and resectability. Patients and surgeon must be prepared for immediate TME after local excision if tumor factors are less than ideal.

### Surgical Technique

Standard transanal excision (TAE) is performed under general or regional anesthesia, often with a pudendal block for analgesia and sphincter relaxation. Retractors or an operating anoscope is used for exposure, a headlamp for visualization, and electrocautery, or other dissecting device, is used to excise a full thickness section of the rectum. The tumor, attached mesorectal fat, and a margin of normal tissue are removed. The rectum is then closed transversely. Practical limits restrict standard TAE to the low rectum.

**Fig. 8:**
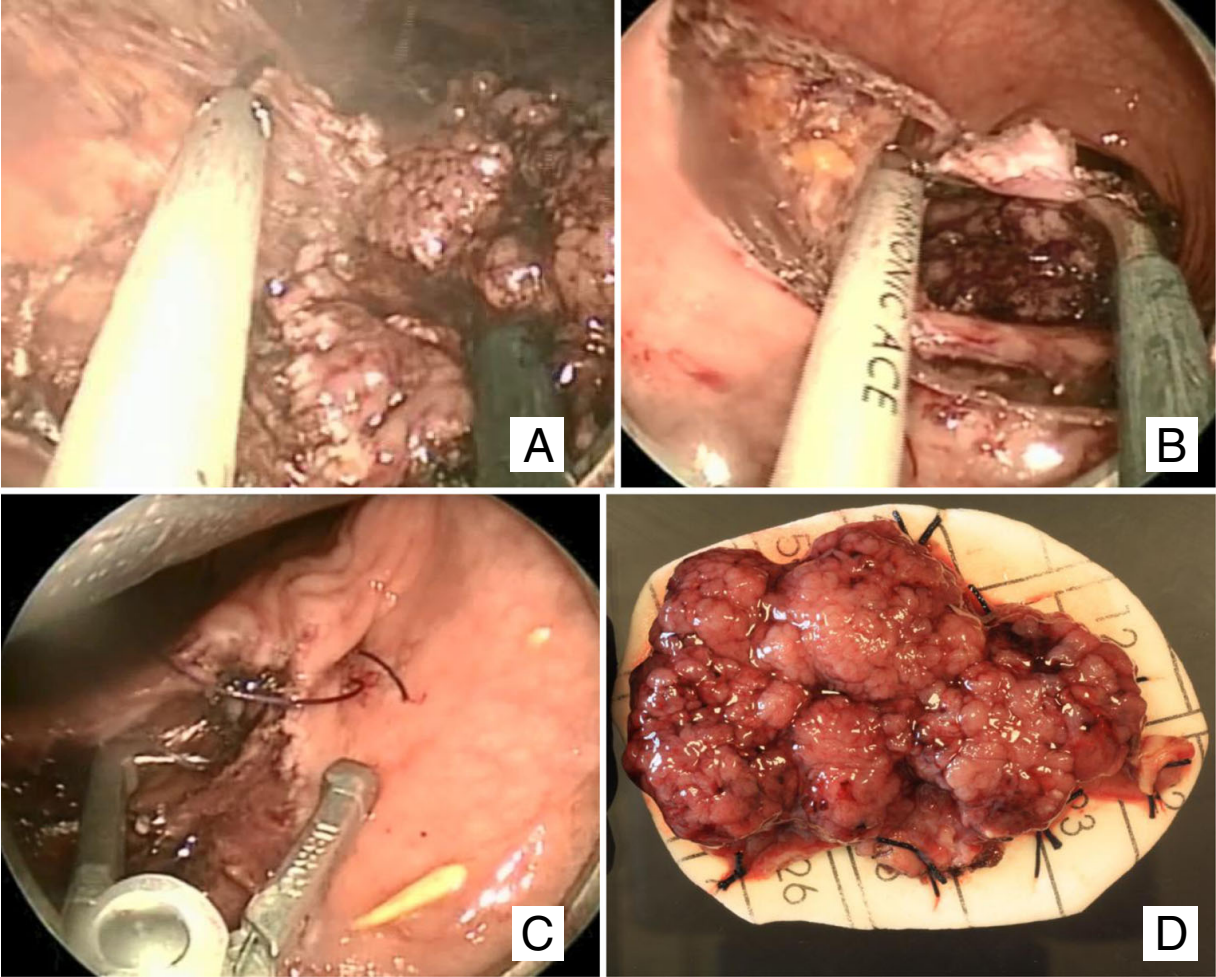
Full thickness local excision of a rectal neoplasm using TEM. **a** Dissection begins with rectal lesion seen at the *right*. **b** Dissection encompassing half the circumference of the rectum, nearly completed. **c** Closure of the defect. **d** Resected specimen, adenoma with a 2-mm focus of T1 adenocarcinoma

TEM and TAMIS provide improved visualization and instrumentation allowing endoluminal excision higher in the rectum. Dr. Gehard Buess of Germany developed and introduced the TEM platform in the 1980s. The platform provides rectal insufflation, 3D visualization and magnification through a stereoscope and binocular eyepiece, and instrumentation via an operating rectoscope. TEM allows for full thickness resection in the extraperitoneal rectum, including perirectal fat and, if necessary, circumferential resection and anastomosis (Fig. 8). In the intraperitoneal rectum, mucosectomy is possible, but full thickness resection will cause loss of rectal insufflation and possibly conversion to abdominal operation.

TAMIS, first described in 2009, is an alternative to TEM for endoluminal resection of rectal lesions. Unlike TEM, which requires specialized, proprietary instrumentation, TAMIS is performed with instruments and techniques familiar to the minimally invasive surgeon. A single incision laparoscopic surgery (SILS) apparatus or gel port-type device serves in place of the TEM platform, and standard laparoscopic surgical instruments are used. Relatively small case series describe the safety and feasibility of TAMIS for resection of adenomas and early rectal cancers,^42^ but the relative infancy of the technique precludes long-term oncologic results at present. TAMIS procedures in the upper rectum^43^ and in combination with transabdominal TME for low rectal cancers^44^ have been described.

### Choice of Technique

Reported outcomes from local excision vary in terms of technique, (neo) adjuvant therapy, and length of follow-up. Few prospective studies exist. After standard TAE, local recurrence has been reported from 0 to 28 % for T1 tumors and overall survival from 74 to 90 %.^45^ You et al.^35^ retrospectively compared the outcomes of 2,124 patients reported to the National Cancer Database and found significantly increased rates of recurrence among patients undergoing local versus standard excision of T1 rectal cancers (12.5 versus 6.9 %, *p* = 0.003), but on multivariate analysis, the type of surgery was not a significant predictor of 5-year overall survival. Local recurrence after TEM has been reported from 0 to 11 % for good risk T1 rectal tumors.^46^ The choice of TAE versus TEM is determined by surgeon preference, equipment availability, and location in the rectum. Tumors in the most distal 5 cm of the rectum can be difficult to access with the TEM platform. Retrospective review of TAE versus TEM patients found increased rates of margin positivity among patients excised with TAE versus TEM, but found similar survival in both groups.^47^ Margin positivity, T stage, distance from the anal verge, but not surgical approach itself were predictors of local recurrence. Tumor factors and quality of resection, therefore, are more important for patient outcome than the operative approach. Proponents of TAMIS argue that cost savings and a shorter learning curve are provided by the more familiar instrumentation. However, ex vivo data suggests that some skills, such as endoscopic sewing, may be more difficult in the TAMIS platform than in TEM.^48^ Furthermore, unlike TEM, a second operator is required to manipulate the laparoscopic camera during TAMIS and TEM equipment may still be necessary in challenging cases, precluding cost savings. Since TAMIS was first described in 2009, no prospective trial comparing its outcomes to TAE or TEM approaches has been completed.

### Extended Indications for Local Excision

Neoadjuvant chemoradiotherapy can extend the utility of local excision to T2N0 tumors. Equivalent disease-free survival at 5 years of follow-up was demonstrated in a randomized clinical trial of chemoradiotherapy plus laparoscopic TME or TEM in 100 patients with T2 rectal cancer.^49^ Although local excision patients had shorter operations and hospitalizations and were less likely to require blood transfusion, early postoperative morbidity and rates of local recurrence and metastasis were not significantly different between the two groups. Other studies have shown equivalent rates of survival with increased rates of local recurrence among patients with T2N0 rectal cancer, although administration of (neo) adjuvant therapy was not uniform in these retrospective studies.^50^^,^ 51 Surgeons and patients must remember that although chemoradiotherapy may extend the indication for local excision to more advanced tumors, it is not without functional consequences. At 1 year after local excision and radiotherapy in a Polish multicenter trial, patients had worse than expected anorectal function, noting that 46 % of patients were incontinent to loose stool and 21 % had significant detriment in global QOL due to anorectal dysfunction. Anorectal functional outcomes were similar to a control group that had undergone anterior resection, although male sexual function was significantly better.^52^ These findings highlight the fact that anorectal function is not solely dependent on surgical preservation of nervous structures and that chemoradiotherapy is not a trivial addition to patient functional outcome.

## Multimodality Therapy and Sphincter Preservation

The goal of neoadjuvant chemotherapy in locally advanced (pT3-T4) rectal cancer is tumor shrinkage—potentially allowing for R_0_ resection and reduced rates of local and distant recurrence. A role for neoadjuvant therapy in increasing rates of sphincter preservation has been hypothesized, either by increasing rates of anterior resection, altering the surgical approach from radical resection to local excision, or adopting a “watch and wait” nonsurgical approach to patients with a complete response to therapy. The ideal regimen for neoadjuvant therapy is a matter of study.^53^ In general, the radiotherapy totals 45–50.4 Gy in 25–28 doses given concomitantly with chemotherapy, which is typically 5-fluorouracil and leucovorin. Other chemotherapy regimens continue to be studied, but no alternative strategy has demonstrated superior results. Surgery is then performed after several weeks, typically 6–8 weeks, although greater intervals may allow for improved tumor response.^54^

It seems a forgone conclusion that if neoadjuvant therapy is efficacious in tumor shrinkage, more patients presumed to require APR would be able to undergo LAR, but data do not support this hypothesis. One trial did demonstrate a 20 % increase in the rate of anterior resection, but this finding was seen only in subgroup analysis and important differences in tumor characteristics were observed between nonradiated and radiated groups.^55^ Meta-analyses of randomized trials, however, do not support increased rates of LAR after chemoradiation. Review of 10 studies revealed no difference in rates of LAR in patients with and without neoadjuvant therapy (*p* = 0.52).^56^ Similarly, a recent Cochrane review of six randomized trials found no effect of neoadjuvant therapy on the rate of sphincter preservation.^57^ At present, the data do not support a role for neoadjuvant therapy in increasing the rate of anterior resection. Additionally, Heald and colleagues have demonstrated an excellent rate of anterior resection (>90 %) with surgery alone, suggesting that patient selection and surgical technique may play more of a role in avoiding APR than does neoadjuvant therapy.

Patients do not respond uniformly to neoadjuvant chemoradiation. An important minority of patients (8–24 %)^58^ have a pathological complete response (pCR), with no viable cancer cells in the resected specimen. Patients with pCR are a unique subset with improved oncologic outcomes and the potential to have organ or sphincter-sparing surgery. Pooled analysis of 484 of these patients from multiple studies revealed that these patients have reduced rates of local recurrence and better overall survival.^58^

Patients with pCR are a subset of those with clinical complete response (cCR). cCR is defined by the absence of endoluminal irregularities on rectal exam or endoscopy, with or without confirmatory radiologic findings on MRI, EUS, or PET/CT. Determining which patients with a cCR also have a pCR is difficult. Radiation-induced inflammation and fibrosis in the rectum and mesorectum render both radiologic and clinical exams unreliable. Many investigators therefore elect to locally excise the previous tumor site after cCR and determine further therapy after re-staging. ypT0-T1 tumors are significantly more likely to have node-negative disease that ypT2-4 tumors (3 versus 39 %, *p* < 0.0001)^59^ and have improved rates of disease-free survival. Issa and colleagues^60^ described 20 patients treated with local excision and found to have pCR. In this study, 49 % of patients with a cCR had a ypT0 tumor at surgery, highlighting the less than perfect predictive value of clinical response. However, ypT0 patients had excellent results, with no recurrences at 87 months of follow-up. Other authors have reported zero recurrence rates for patients with significant downstaging treated with local excision.^61^ While ypT0 tumors may be definitively treated by local excision, the optimal management of patients with ypT1 or ypT2 disease may be proctectomy rather than local excision. Perez and colleagues described 33 patients with small residual clinical disease resected with TEM after neoadjuvant therapy. Fifteen percent recurred, exclusively within the ypT1-T2 group. Disease-free survival at 12 months was 68 %.^62^

The Brazilian group led by Habr-Gama is at the forefront of complete nonoperative management for cCR patients. Most recently, they described 70 rectal cancer patients undergoing neoadjuvant CRT: 54 Gy of radiation and 6 cycles of 5-FU/leucovorin, with 68 % having a complete clinical response at 10 weeks following the completion of radiotherapy. These patients were followed with clinical exam and either MRI or PET/CT every 2 months for the first year, with less frequent but regular follow-up thereafter. Patients without irregularities were managed nonoperatively, those with subtle irregularities underwent TEM, and those with pathologic or gross tumor recurrence underwent radical surgery. At 1 year, 57 % sustained complete response, and 51 % were free of recurrence at a median follow-up of 56 months.^63^ These results indicate that a small, but important group of rectal cancer patients with complete response to neoadjuvant therapy can perhaps be managed nonoperatively, but these data need to be replicated at other centers.

Neoadjuvant therapy is the standard of care for patients with locally advanced rectal cancers. Selected patients with excellent clinical or pathological response to neoadjuvant therapy may be candidates for organ preservation within the context of a highly rigorous follow-up program. However, chemoradiation has not been proven to increase rates of anterior resection or sphincter preservation among locally advanced rectal cancer patients as a group. Subject for ongoing study and debate include predicting how tumors will respond to neoadjuvant therapy, monitoring patients for response to therapy, the optimal neoadjuvant regimen, and determining the optimal treatment for patients with excellent, but incomplete, response to neoadjuvant therapy.

## The Role of the APR in the Era of Sphincter-Saving Techniques

**Fig. 9:**
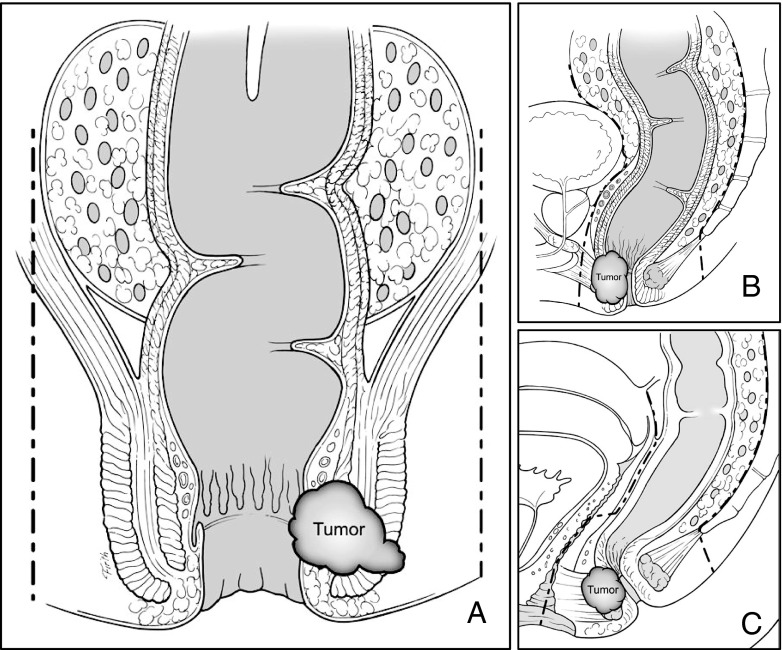
Abdominoperineal resection. **a** Anterior view demonstrating abdominal dissection in TME plane with perineal specimen including sphincter complex for a very low rectal tumor. **b** Lateral view of resection planes in the male. **c** Lateral view of resection planes in the female

The indications for APR have changed dramatically since it was first described by Miles in the mid-twentieth century. Sphincter preservation is now viewed as a mark of quality in rectal cancer surgery. Oncologic outcomes, not just QOL metrics, have also raised concerns that APR is an inferior operation compared to anterior resection. Dissection close to the rectum at the level of the puborectalis often creates a specimen “waist” and yields positive CRMs. Pooled analysis of 14 European rectal cancer studies found positive CRMs in 10 and 5 % of APR and anterior resection specimens, respectively.^64^ Local recurrence rates were significantly elevated (20 versus 11 %) and 5-year overall survival worse (59 versus 70 %) in patients with an APR as compared to an LAR.^64^ These inferior outcomes in patients after APR could be due to deficiencies in the surgical technique and/or tumor characteristics. A review of 24 high quality studies found APR to be associated with higher rates of CRM positivity and tumor perforation and inferior oncologic outcomes, but also found tumors in patients with APRs to be lower and more locally advanced.^65^ Dramatic reduction in local recurrence rates and margin positivity can be achieved by improving and standardizing the perineal phase of surgery. Appropriate extralevator resection planes follow the deep perineal fasica to the levators which are then transected to connect with the mesorectum (Fig. 9). As with transabdominal TME, care is taken to avoid “waisting of the specimen.” Martijinse and colleagues^66^ found that introduction of a quality improvement program focusing on extralevator perineal dissection reduced the rate of R_1_ resections of T4 tumors from 30.2 to 5.7 %. Focus on performance improvement, improved imaging, and adoption of neoadjuvant therapy on a national level in the Netherlands have recently been shown to negate the difference in CRM positivity between LAR and APR in that country.^67^ Therefore, the patient requiring an APR, even for a locally advanced tumor, may also have a comparable oncologic outcome to a patient undergoing anterior resection.

Even in the era of sphincter-sparing surgery, APR is still the operation of choice for patients with very low tumors or poor preoperative function. Tumor characteristics necessitating an APR include a T3 or T4 tumor in the anal canal or involving the levator ani/external sphincter muscle. Patients with impaired continence; previous obstetric, traumatic, or iatrogenic injury to the sphincters; or chronic diarrheal diseases such as inflammatory bowel diseases are likely to do poorly following creation of a low anastomosis. Patients should be counseled about the likelihood of postoperative defecatory problems, and any patient unable to accept these potential issues should undergo APR. A patient’s preference for a stoma rather than incontinence should certainly not be discounted, even in a potential candidate for anterior resection.

## Maintaining Intestinal Continuity After APR

### Selected Patients Undergoing APRs May Be Able to Maintain Intestinal Continuity

Several authors have described restoration of intestinal continuity following APR. Perineal colostomy, graciloplasty, and artificial sphincters provide pseudocontinence: intestinal continuity is maintained, but antegrade or retrograde colonic enemas may be required for defecation. Even though an immediate reconstruction at the time of APR could technically be performed, it is generally discouraged in favor of a secondary approach after the oncological long-term goals have been met. Parenthetically, the post-APR abdominoperineal reconstruction is typically not covered by any insurance. Unlike ISR, for which many large series are available for comparison, data on oncologic and functional outcomes for pseudocontinent procedures is sparse, often limited to highly specialized centers.

One option for a perineal colostomy restores intestinal continuity and creates pseudocontinence using a band of autologous colonic tissue. Two centimeters proximal to the skin, a 10-cm long strip of colonic tissue is folded on itself, wrapped around the neorectum, and secured in order that this band might provide a measure of continence for the perineal colostomy. Colonic irrigations are performed for defecation. Lasser and colleagues reported on 40 patients who underwent this procedure following APR for rectal cancer.^68^ Morbidity was high (55 %) with eight patients suffering perineal suppuration. Four patients underwent conversion to colostomy—two due to complications and two due to functional failures. Of the remaining patients, only four were perfectly continent.^69^ Comparison of small numbers of patients after ISR (*n* = 14) and after psuedocontinent perineal colostomy (*n* = 22) failed to find significant differences in QOL and continence between the two groups.^70^

Graciloplasty, either single or double, allows for the reconstruction of a neosphincter following APR but is often followed by muscle fatigue and poor function. Dynamic graciloplasty is achieved by the inclusion of a subcutaneous pulse generator, though the use of the generator in the USA would be off label because of lack of FDA approval. As in patients whose neosphincter was formed with autologous colonic tissues, graciloplasty patients would need to perform colonic irrigation with either retrograde or antegrade enemas (with the creation of a Malone appendicostomy). A recent study of 10 patients undergoing dynamic graciloplasty with Malone appendicostomy after APR reported high morbidity with 90 % requiring some type of reoperation.^71^ Despite the high complication rates, many patients may ultimately achieve satisfactory functional outcomes.^72^

A third described option to enhance function after APR is through the use of the artificial bowel sphincter (ABS). The ABS consists of a subcutaneous cuff surrounding the anus and a pump in the labial or scrotal area. Patients are trained to inflate and deflate the cuff in order to achieve defecation. Report of eight patients undergoing ABS placement in a synchronous or a delayed fashion after APR demonstrated that, although a significant learning curve existed for the patients, all but one were able to achieve a good continence score.^73^ Three patients had early impaired defecation and one suffered a wound infection.

Restoration of intestinal continuity after APR is a challenge and the available procedures to achieve pseudocontinence are limited by relatively little experience and, in some cases, high rates of morbidity. However, these procedures may be appropriate for well-informed, highly motivated patients who are not candidates for anterior or intersphincteric resection and want to avoid a stoma at all costs.

## Conclusion

Surgical therapy for rectal cancer has, over the past 100 years, evolved from the radical operation of Miles to a number of surgical options and novel techniques. These changes have been driven by increased understanding of the pathophysiology of rectal cancer, multimodality treatment, improved technology, surgical innovation, and by surgeons placing greater emphasis on the patient’s QOL. As a result of this progress, the postoperative patient with rectal cancer not only has an improved chance of survival, but has real potential for continence and normal sexual and urinary function. In the coming years, relatively new procedures such as ISR, APPEAR, and transanal TME will test the limits of what can be achieved with sphincter-sparing resections for low rectal cancers, while advances in multidisciplinary treatment may extend the ability of surgeons to minimize surgical intervention. Regardless of technique, however, the primary goal of surgical treatment of rectal cancer remains oncologic cure, while preserving sphincter function and maintaining intestinal continuity remain secondary goals.

## References

[1] Lange MM, Rutten HJ, van de Velde CJ. One hundred years of curative surgery for rectal cancer: 1908–2008. Eur J Surg Oncol. 2009 May;35(5):456-63. 10.1016/j.ejso.2008.09.012 19013050

[2] Miles WE. Cancer of the rectum. Lettsomian lectures. London; 1923.

[3] Dixon CF. Anterior Resection for Malignant Lesions of the Upper Part of the Rectum and Lower Part of the Sigmoid. Ann Surg. 1948 Sep; 128(3):425-42. 10.1097/00000658-194809000-00009 PMC1514072 17859211

[4] Ricciardi R, Virnig BA, Madoff RD, Rothenberger DA, Baxter NN. The status of radical proctectomy and sphincter-sparing surgery in the United States. Dis Colon Rectum. 2007 Aug; 50(8): 1119-27. 10.1007/s10350-007-0250-5 17573548

[5] Williams NS, Dixon MF, Johnston D. Reappraisal of the 5 centimetre rule of distal excision for carcinoma of the rectum: a study of distal intramural spread and of patients’ survival. Br J Surg. 1983 Mar; 70(3):150-4. 10.1002/bjs.1800700305 6831156

[6] Shirouzu K, Isomoto H, Kakegawa T. Distal spread of rectal cancer and optimal distal margin of resection for sphincter-preserving surgery. Cancer. 1995 Aug 1; 76(3):388-92. 10.1002/1097-0142(19950801)76:3<388::aid-cncr2820760307>3.0.co;2-y 8625118

[7] Pollett WG, Nicholls RJ. The relationship between the extent of distal clearance and survival and local recurrence rates after curative anterior resection for carcinoma of the rectum. Ann Surg. 1983 Aug; 198(2):159-63. 10.1097/00000658-198308000-00008 PMC1353073 6870373

[8] Leo E, Belli F, Miceli R, Mariani L, Gallino G, Battaglia L, Vannelli A, Andreola S. Distal clearance margin of 1 cm or less: a safe distance in lower rectum cancer surgery. Int J Colorectal Dis. 2009 Mar;24(3):317-22. 10.1007/s00384-008-0604-z 18931846

[9] Bujko K, Rutkowski A, Chang GJ, Michalski W, Chmielik E, Kusnierz J. Is the 1-cm rule of distal bowel resection margin in rectal cancer based on clinical evidence? A systematic review. Ann Surg Oncol. 2012 Mar;19(3):801-8. 10.1245/s10434-011-2035-2 PMC3278608 21879269

[10] Quirke P, Durdey P, Dixon MF, Williams NS. Local recurrence of rectal adenocarcinoma due to inadequate surgical resection. Histopathological study of lateral tumour spread and surgical excision. Lancet. 1986 Nov 1;2(8514):996-9. 10.1016/s0140-6736(86)92612-7 2430152

[11] Adam IJ, Mohamdee MO, Martin IG, Scott N, Finan PJ, Johnston D, Dixon MF, Quirke P. Role of circumferential margin involvement in the local recurrence of rectal cancer. Lancet. 1994 Sep 10;344(8924):707-11. 10.1016/s0140-6736(94)92206-3 7915774

[12] Heald RJ, Ryall RD. Recurrence and survival after total mesorectal excision for rectal cancer. Lancet. 1986 Jun 28;1(8496):1479-82. 10.1016/s0140-6736(86)91510-2 2425199

[13] Marr R, Birbeck K, Garvican J, Macklin CP, Tiffin NJ, Parsons WJ, Dixon MF, Mapstone NP, Sebag-Montefiore D, Scott N, Johnston D, Sagar P, Finan P, Quirke P. The modern abdominoperineal excision: the next challenge after total mesorectal excision. Ann Surg. 2005 Jul;242(1):74-82. 10.1097/01.sla.0000167926.60908.15 PMC1357707 15973104

[14] Martling AL, Holm T, Rutqvist LE, Moran BJ, Heald RJ, Cedemark B. Effect of a surgical training programme on outcome of rectal cancer in the County of Stockholm. Stockholm Colorectal Cancer Study Group, Basingstoke Bowel Cancer Research Project. Lancet. 2000 Jul 8; 356(9224):93-6. 10.1016/s0140-6736(00)02469-7 10963244

[15] Heald RJ. Towards fewer colostomies—the impact of circular stapling devices on the surgery of rectal cancer in a district hospital. Br J Surg. 1980 Mar; 67(3):198-200. 10.1002/bjs.1800670311 7362961

[16] Heald RJ. “Rectal Cancer in the 21st Century—Radical Operations: Anterior Resection and Abdominoperineal Excision.” *Mastery of Surgery, 5th Edition.* Ed. Josef E. Fischer. Philadelphia: Lippincott, Williams & Wilkins, 2007. 1544-1555.

[17] Heald RJ, Moran BJ, Ryall RD, Sexton R, MacFarlane JK. Rectal cancer: the Basingstoke experience of total mesorectal excision, 1978–1997. Arch Surg. 1998 Aug;133(8):894-9. 10.1001/archsurg.133.8.894 9711965

[18] Piso P, Dahlke MH, Mirena P, Schmidt U, Aselmann H, Schlitt HJ, Raab R, Klempnauer J. Total mesorectal excision for middle and lower rectal cancer: a single institution experience with 337 consecutive patients. J Surg Oncol. 2004 Jun 1;86(3):115-21. 10.1002/jso.20062 15170648

[19] Bryant CL, Lunniss PJ, Knowles CH, Thaha MA, Chan CL. Anterior resection syndrome. Lancet Oncol. 2012 Sep;13(9):e403-8. 10.1016/S1470-2045(12)70236-X 22935240

[20] Lundby L, Krogh K, Jensen VJ, Gandrup P, Qvist N, Overgaard J, Laurberg S. Long-term anorectal dysfunction after postoperative radiotherapy for rectal cancer. Dis Colon Rectum. 2005 Jul;48(7):1343-9. 10.1007/s10350-005-0049-1 15933797

[21] Lee SJ, Park YS. Serial evaluation of anorectal function following low anterior resection of the rectum. Int J Colorectal Dis. 1998;13(5-6):241-6. 10.1007/s003840050169 9870169

[22] Wallner C, Lange MM, Bonsing BA, Maas CP, Wallace CN, Dabhoiwala NF, Rutten HJ, Lamers WH, Deruiter MC, van de Velde CJ; Cooperative Clinical Investigators of the Dutch Total Mesorectal Excision Trial. Causes of fecal and urinary incontinence after total mesorectal excision for rectal cancer based on cadaveric surgery: a study from the Cooperative Clinical Investigators of the Dutch total mesorectal excision trial. J Clin Oncol. 2008 Sep 20;26(27):4466-72. 10.1200/JCO.2008.17.3062 18802159

[23] Schiessel R, Karner-Hanusch J, Herbst F, Teleky B, Wunderlich M. Intersphincteric resection for low rectal tumours. Br J Surg. 1994 Sep;81(9):1376-8. 10.1002/bjs.1800810944 7953423

[24] Kuo LJ, Hung CS, Wang W, Tam KW, Lee HC, Liang HH, Chang YJ, Huang MT, Wei PL. Intersphincteric resection for very low rectal cancer: clinical outcomes of open versus laparoscopic approach and multidimensional analysis of the learning curve for laparoscopic surgery. J Surg Res. 2013 Aug;183(2):524-30. 10.1016/j.jss.2013.01.049 23465434

[25] Baek SJ, Al-Asari S, Jeong DH, Hur H, Min BS, Baik SH, Kim NK. Robotic versus laparoscopic coloanal anastomosis with or without intersphincteric resection for rectal cancer. Surg Endosc. 2013 Nov;27(11):4157-63. 10.1007/s00464-013-3014-4 23708725

[26] Schiessel R, Novi G, Holzer B, Rosen HR, Renner K, Hölbling N, Feil W, Urban M. Technique and long-term results of intersphincteric resection for low rectal cancer. Dis Colon Rectum. 2005 Oct;48(10):1858-65. 10.1007/s10350-005-0134-5 16086223

[27] Martin ST, Heneghan HM, Winter DC. Systematic review of outcomes after intersphincteric resection for low rectal cancer. Br J Surg. 2012 May;99(5):603-12. 10.1002/bjs.8677 22246846

[28] Renner K, Rosen HR, Novi G, Hölbling N, Schiessel R. Quality of life after surgery for rectal cancer: do we still need a permanent colostomy? Dis Colon Rectum. 1999 Sep;42(9):1160-7. 10.1007/BF02238568 10496556

[29] Rullier E, Sa Cunha A, Couderc P, Rullier A, Gontier R, Saric J. Laparoscopic intersphincteric resection with coloplasty and coloanal anastomosis for mid and low rectal cancer. Br J Surg. 2003 Apr;90(4):445-51. 10.1002/bjs.4052 12673746

[30] Denost Q, Laurent C, Capdepont M, Zerbib F, Rullier E. Risk factors for fecal incontinence after intersphincteric resection for rectal cancer. Dis Colon Rectum. 2011 Aug;54(8):963-8. 10.1097/DCR.0b013e31821d3677 21730784

[31] Williams NS, Murphy J, Knowles CH. Anterior Perineal PlanE for Ultra-low Anterior Resection of the Rectum (the APPEAR technique): a prospective clinical trial of a new procedure. Ann Surg. 2008 May;247(5):750-8. 10.1097/SLA.0b013e31816b2ee3 18438111

[32] Sylla P, Bordeianou LG, Berger D, Han KS, Lauwers GY, Sahani DV, Sbeih MA, Lacy AM, Rattner DW. A pilot study of natural orifice transanal endoscopic total mesorectal excision with laparoscopic assistance for rectal cancer. Surg Endosc. 2013 Sep;27(9):3396-405 10.1007/s00464-013-2922-7 23572214

[33] de Lacy AM, Rattner DW, Adelsdorfer C, Tasende MM, Fernández M, Delgado S, Sylla P, Martínez-Palli G. Transanal natural orifice transluminal endoscopic surgery (NOTES) rectal resection: “down-to-up” total mesorectal excision (TME)—short-term outcomes in the first 20 cases. Surg Endosc. 2013 Sep;27(9):3165-72. 10.1007/s00464-013-2872-0 23519489

[34] Emhoff IA, Lee GC, Sylla P. Transanal colorectal resection using natural orifice translumenal endoscopic surgery (NOTES). Dig Endosc. 2013 Aug 28. 10.1111/den.12157 24033375

[35] You YN, Baxter NN, Stewart A, Nelson H. Is the increasing rate of local excision for stage I rectal cancer in the United States justified?: a nationwide cohort study from the National Cancer Database. Ann Surg. 2007 May;245(5):726-33. 10.1097/01.sla.0000252590.95116.4f PMC1877081 17457165

[36] Puli SR, Reddy JB, Bechtold ML, Choudhary A, Antillon MR, Brugge WR. Accuracy of endoscopic ultrasound to diagnose nodal invasion by rectal cancers: a meta-analysis and systematic review. Ann Surg Oncol. 2009 May;16(5):1255-65. 10.1245/s10434-009-0337-4 19219506

[37] Puli SR, Bechtold ML, Reddy JB, Choudhary A, Antillon MR, Brugge WR. How good is endoscopic ultrasound in differentiating various T stages of rectal cancer? Meta-analysis and systematic review. Ann Surg Oncol. 2009 Feb;16(2):254-65. 10.1245/s10434-008-0231-5 19018597

[38] Al-Sukhni E, Milot L, Fruitman M, Beyene J, Victor JC, Schmocker S, Brown G, McLeod R, Kennedy E. Diagnostic accuracy of MRI for assessment of T category, lymph node metastases, and circumferential resection margin involvement in patients with rectal cancer: a systematic review and meta-analysis. Ann Surg Oncol. 2012 Jul;19(7):2212-23. 10.1245/s10434-011-2210-5 22271205

[39] Bach SP, Hill J, Monson JR, Simson JN, Lane L, Merrie A, Warren B, Mortensen NJ; Association of Coloproctology of Great Britain and Ireland Transanal Endoscopic Microsurgery (TEM) Collaboration. A predictive model for local recurrence after transanal endoscopic microsurgery for rectal cancer. Br J Surg. 2009 Mar;96(3):280-90 10.1002/bjs.6456 19224520

[40] Borschitz T, Heintz A, Junginger T. The influence of histopathologic criteria on the long-term prognosis of locally excised pT1 rectal carcinomas: results of local excision (transanal endoscopic microsurgery) and immediate reoperation. Dis Colon Rectum. 2006 Oct;49(10):1492-506; discussion 1500-5. 10.1007/s10350-006-0587-1 16897336

[41] Hahnloser D, Wolff BG, Larson DW, Ping J, Nivatvongs S. Immediate radical resection after local excision of rectal cancer: an oncologic compromise? Dis Colon Rectum. 2005 Mar;48(3):429-37. 10.1007/s10350-004-0900-9 15747069

[42] Albert MR, Atallah SB, deBeche-Adams TC, Izfar S, Larach SW. Transanal minimally invasive surgery (TAMIS) for local excision of benign neoplasms and early-stage rectal cancer: efficacy and outcomes in the first 50 patients. Dis Colon Rectum. 2013 Mar;56(3):301-7. 10.1097/DCR.0b013e31827ca313 23392143

[43] Hussein Q, Artinyan A. Pushing the Limits of Local Excision for Rectal Cancer: Transanal Minimally Invasive Surgery for an Upper Rectal/Rectosigmoid Lesion. Ann Surg Oncol. 2014 Jan 10. 10.1245/s10434-013-3457-9 24407315

[44] Atallah S, Albert M, DeBeche-Adams T, Nassif G, Polavarapu H, Larach S. Transanal minimally invasive surgery for total mesorectal excision (TAMIS-TME): a stepwise description of the surgical technique with video demonstration. Tech Coloproctol. 2013 Jun;17(3):321-5. 10.1007/s10151-012-0971-x 23377536

[45] Perretta S, Guerrero V, Garcia-Aguilar J. Surgical treatment of rectal cancer: local resection. Surg Oncol Clin N Am. 2006 Jan;15(1):67-93 10.1016/j.soc.2005.10.001 16389151

[46] Kunitake H, Abbas MA. Transanal endoscopic microsurgery for rectal tumors: a review. Perm J. 2012 Spring;16(2):45-50. 10.7812/tpp/11-120 PMC3383161 22745615

[47] Christoforidis D, Cho HM, Dixon MR, Mellgren AF, Madoff RD, Finne CO. Transanal endoscopic microsurgery versus conventional transanal excision for patients with early rectal cancer. Ann Surg. 2009 May;249(5):776-82. 10.1097/SLA.0b013e3181a3e54b 19387326

[48] Rimonda R, Arezzo A, Arolfo S, Salvai A, Morino M. TransAnal Minimally Invasive Surgery (TAMIS) with SILS™ port versus Transanal Endoscopic Microsurgery (TEM): a comparative experimental study. Surg Endosc. 2013 Oct;27(10):3762-8. 10.1007/s00464-013-2962-z 23636523

[49] Lezoche E, Baldarelli M, Lezoche G, Paganini AM, Gesuita R, Guerrieri M. Randomized clinical trial of endoluminal locoregional resection versus laparoscopic total mesorectal excision for T2 rectal cancer after neoadjuvant therapy. Br J Surg. 2012 Sep;99(9):1211-8. 10.1002/bjs.8821 22864880

[50] Allaix ME, Arezzo A, Giraudo G, Morino MJ. Transanal endoscopic microsurgery vs. laparoscopic total mesorectal excision for T2N0 rectal cancer. Gastrointest Surg. 2012 Dec;16(12):2280-7. 10.1007/s11605-012-2046-8 23070621

[51] Lee W, Lee D, Choi S, Chun H. Transanal endoscopic microsurgery and radical surgery for T1 and T2 rectal cancer. Surg Endosc. 2003 Aug;17(8):1283-7. 10.1007/s00464-002-8814-x 12739119

[52] Gornicki A, Richter P, Polkowski W, Szczepkowski M, Pietrzak L, Kepka L, Rutkowski A, Bujko K. Anorectal and sexual functions after preoperative radiotherapy and full-thickness local excision of rectal cancer. Eur J Surg Oncol. 2013 Dec 4. pii: S0748-7983(13)00918-9. 10.1016/j.ejso.2013.11.010 24332947

[53] Baker B, Salameh H, Al-Salman M, and Daoud F. How does preoperative radiotherapy affect the rate of sphincter-sparing surgery in rectal cancer? Surgical Oncology, 2012 Sep; 21 (3): e103-e109. 10.1016/j.suronc.2012.03.004 22534311

[54] Petrelli F, Sgroi G, Sarti E, Barni S. Increasing the Interval Between Neoadjuvant Chemoradiotherapy and Surgery in Rectal Cancer: A Meta-Analysis of Published Studies. Ann Surg. 2013 Nov 20. [Epub]. 10.1097/SLA.0000000000000368 24263329

[55] R. Sauer, H. Becker, W. Hohenberger et al. Preoperative versus postoperative chemoradiotherapy for rectal cancer. N Engl J Med. 2004; 351:1731–1740. 10.1056/NEJMoa040694 15496622

[56] Bujko K, Kepka L, Michalski W, Nowacki MP. Does rectal cancer shrinkage induced by preoperative radio (chemo) therapy increase the likelihood of anterior resection? A systematic review of randomised trials. Radiother Oncol. 2006 Jul;80(1):4-12. 10.1016/j.radonc.2006.04.012 16730086

[57] McCarthy K, Pearson K, Fulton R, Hewitt J. Pre-operative chemoradiation for non-metastatic locally advanced rectal cancer. Cochrane Database Syst Rev. 2012 Dec 12;12:CD008368. 10.1002/14651858.CD008368.pub2 PMC12015662 23235660

[58] Maas M, Nelemans PJ, Valentini V, Das P, Rödel C, Kuo LJ, Calvo FA, García-Aguilar J, Glynne-Jones R, Haustermans K, Mohiuddin M, Pucciarelli S, Small W Jr, Suárez J, Theodoropoulos G, Biondo S, Beets-Tan RG, Beets GL. Long-term outcome in patients with a pathological complete response after chemoradiation for rectal cancer: a pooled analysis of individual patient data. Lancet Oncol. 2010 Sep;11(9):835-44. 10.1016/S1470-2045(10)70172-8 20692872

[59] Read TE, Andujar JE, Caushaj PF, Johnston DR, Dietz DW, Myerson RJ, Fleshman JW, Birnbaum EH, Mutch MG, Kodner IJ. Neoadjuvant therapy for rectal cancer: histologic response of the primary tumor predicts nodal status. Dis Colon Rectum. 2004 Jun;47(6):825-31. 10.1007/s10350-004-0535-x 15108025

[60] Issa N, Murninkas A, Powsner E, Dreznick Z. Long-term outcome of local excision after complete pathological response to neoadjuvant chemoradiation therapy for rectal cancer. World J Surg. 2012 Oct;36(10):2481-7. 10.1007/s00268-012-1697-7 22736345

[61] Schell SR, Zlotecki RA, Mendenhall WM, Marsh RW, Vauthey JN, Copeland EM 3^rd^. Transanal excision of locally advanced rectal cancers downstaged using neoadjuvant chemoradiotherapy. J Am Coll Surg. 2002 May;194(5):584-90; discussion 590-1. 10.1016/s1072-7515(02)01128-6 12025835

[62] Perez RO, Habr-Gama A, Lynn PB, São Julião GP, Bianchi R, Proscurshim I, Gama-Rodrigues J. Transanal endoscopic microsurgery for residual rectal cancer (ypT0-2) following neoadjuvant chemoradiation therapy: another word of caution. Dis Colon Rectum. 2013 Jan;56(1):6-13. 10.1097/DCR.0b013e318273f56f 23222274

[63] Habr-Gama A, Sabbaga J, Gama-Rodrigues J, São Julião GP, Proscurshim I, Bailão Aguilar P, Nadalin W, Perez RO. Watch and wait approach following extended neoadjuvant chemoradiation for distal rectal cancer: are we getting closer to anal cancer management? Dis Colon Rectum. 2013 Oct;56(10):1109-17. 10.1097/DCR.0b013e3182a25c4e 24022527

[64] den Dulk M, Putter H, Collette L, Marijnen CA, Folkesson J, Bosset JF, Rödel C, Bujko K, Påhlman L, van de Velde CJ. The abdominoperineal resection itself is associated with an adverse outcome: the European experience based on a pooled analysis of five European randomized clinical trials on rectal cancer. Eur J Cancer. 2009 May;45(7):1175-83. 10.1016/j.ejca.2008.11.039 19128956

[65] How P, Shihab O, Tekkis P, Brown G, Quirke P, Heald R, Moran B. A systematic review of cancer related patient outcomes after anterior resection and abdominoperineal excision for rectal cancer in the total mesorectal excision era. Surg Oncol. 2011 Dec;20(4):e149-55. 10.1016/j.suronc.2011.05.001 21632237

[66] Martijnse IS, Dudink RL, West NP, Wasowicz D, Nieuwenhuijzen GA, van Lijnschoten I, Martijn H, Lemmens VE, van de Velde CJ, Nagtegaal ID, Quirke P, Rutten HJ. Focus on extralevator perineal dissection in supine position for low rectal cancer has led to better quality of surgery and oncologic outcome. Ann Surg Oncol. 2012 Mar;19(3):786-93. 10.1245/s10434-011-2004-9 21861224

[67] van Leersum N, Martijnse I, den Dulk M, Kolfschoten N, Le Cessie S, van de Velde C, Tollenaar R, Wouters M, Rutten HJ. Differences in Circumferential Resection Margin Involvement After Abdominoperineal Excision and Low Anterior Resection No Longer Significant. Ann Surg. 2013 Oct 3 10.1097/SLA.0000000000000225 24096756

[68] Lasser P, Dubé P, Guillot JM, Elias D. Pseudocontinent perineal colostomy following abdominoperineal resection: technique and findings in 49 patients. Eur J Surg Oncol. 2001 Feb;27(1):49-53. 10.1053/ejso.2000.1046 11237492

[69] Marchal F, Doucet C, Lechaux D, Lasser P, Lehur PA. Secondary implantation of an artificial sphincter after abdominoperineal resection and pseudocontinent perineal colostomy for rectal cancer. Gastroenterol Clin Biol. 2005 Apr;29(4):425-8. 10.1016/s0399-8320(05)80797-3 15864207

[70] Dumont F, Ayadi M, Goéré D, Honoré C, Elias D. Comparison of fecal continence and quality of life between intersphincteric resection and abdominoperineal resection plus perineal colostomy for ultra-low rectal cancer. J Surg Oncol. 2013 Sep;108(4):225-9. 10.1002/jso.23379 23868337

[71] Abbes Orabi N, Vanwymersch T, Paterson HM, Mauel E, Jamart J, Crispin B, Kartheuser A. Total perineal reconstruction after abdominoperineal excision for rectal cancer: long-term results of dynamic graciloplasty with Malone appendicostomy. Colorectal Dis. 2011 Apr;13(4):406-13. 10.1111/j.1463-1318.2009.02168.x 20041927

[72] Violi V, Boselli AS, De Bernardinis M, Costi R, Nervi G, Bertelè A, Franzè A, Roncoroni L. Surgical results and functional outcome after total anorectal reconstruction by double graciloplasty supported by external-source electrostimulation and/or implantable pulse generators: an 8-year experience. Int J Colorectal Dis. 2004 May;19(3):219-27. 10.1007/s00384-003-0528-6 14586631

[73] Romano G, La Torre F, Cutini G, Bianco F, Esposito P, Montori A. Total anorectal reconstruction with the artificial bowel sphincter: report of eight cases. A quality-of-life assessment. Dis Colon Rectum. 2003 Jun;46(6):730-4. 10.1007/s10350-004-6649-3 12794573

